# Short-Range Water Temperature Profiling in a Lake with Coastal Acoustic Tomography

**DOI:** 10.3390/s20164498

**Published:** 2020-08-12

**Authors:** Haocai Huang, Yong Guo, Guangming Li, Kaneko Arata, Xinyi Xie, Pan Xu

**Affiliations:** 1 Ocean College, Zhejiang University, Zhoushan 316021, China; hchuang@zju.edu.cn (H.H.); guo_yong@zju.edu.cn (Y.G.); 21934184@zju.edu.cn (X.X.); 2 National Innovation Institute of Defense Technology, Fengtai District, Beijing 100071, China; 3 Graduate School of Engineering, Hiroshima University, Higashi-Hiroshima, Hiroshima 739-8527, Japan; aratak@hiroshima-u.ac.jp; 4 College of Meteorology and Oceanography, National University of Defense Technology, Kaifu District, Changsha 410073, China; xupan09@nudt.edu.cn

**Keywords:** coastal acoustic tomography, vertical slice inversion, multi-path arrival identification, position correction, water temperature observation

## Abstract

Coastal acoustic tomography (CAT), as an innovative technology, can perform water temperature measurements both in horizontal and vertical slices. Investigations on vertical slice observations are significantly fewer in number than horizontal observations due to difficulties in multi-path arrival peak identification. In this study, a two-station sound transmission experiment is carried out in Thousand-Island Lake, Hangzhou, China, to acquire acoustic data for water temperature profiling. Time windows, determined by range-independent ray simulation, are used to identify multi-path arrival peaks and obtain corresponding sound wave travel times. Special attention is paid to travel time correction, whose errors are caused by position drifting by more than 2 m of moored stations. The sound speed and temperature profiling are divided into four layers and are calculated by regularized inversion. Results show a good consistency with conductivity–temperature–depth (CTD) measurements. The root mean square error (RMSE) of water temperature is 0.3494, 0.6838, 1.0236 and 1.0985 °C for layer 1, 2, 3 and 4, respectively. The fluctuations of measurement are further smoothed by the moving average, which decreases the RMSE of water temperature to 0.2858, 0.4742, 0.7719 and 0.9945 °C, respectively. This study illustrates the feasibility and high accuracy of the coastal acoustic tomography method in short-range water temperature measurement. Furthermore, 3D water temperature field profiling can be performed with combined analyzing in horizontal and vertical slices.

## 1. Introduction

Water temperature can be measured by fixed-point sensors such as temperature chain, thermocouple and other in situ measuring devices, which is time consuming with distribution measurement [1]. Water temperature of a surface layer can also be sensed by remote sensing methods such as radar and satellite [2], while distribution and variation progress of water columns cannot be monitored using those technique. Ocean acoustic tomography (OAT) is a powerful technology that can map the internal distribution structures of water parameters [3,4,5], and many experiments have demonstrated the effectiveness of OAT in the past few years for flow current profiling and internal tide and sound speed measuring [6,7,8]. Coastal acoustic tomography (CAT) is an innovative technology to monitor water temperature variations in the coastal area. It was developed as a coastal application of the ocean acoustic tomography technique [9]. CAT can conduct real-time field observations with multi-station networking, which is a prominent advantage in comparison with fixed-point and sensor array measurements [10,11,12,13,14]. A significant number of experiments have been conducted to measure range-average temperatures or map temperature distribution vertically and horizontally with CAT [15,16,17,18]. However, few researchers have attempted the short-range temperature observation, which may be used to monitor specific targets in small ranges, such as artificial upwelling and hydrothermal areas [15,19]. A higher frequency sound wave is needed for a high requirement of time resolution in short-range acoustic tomography research. Besides, vertical slice inversion, which requires the multi-path arrival signal identification of the sound transmission, has not been sufficiently studied until now. Existing research only identifies very few arrival peaks or conducts experiments within laboratories [17,20], mainly due to the significant transmission loss caused by sound scattering after the bottom reflection and low resolution of the transmitted signal. Chen et al. successfully identified three arrival peaks using CATs enabled with mirror-transpond functionality [21], but no further study regarding vertical slice measurements is reported.

In this study, a two-station sound transmission experiment was conducted in Thousand-Island Lake, Hangzhou, China, aiming to test the feasibility and accuracy of CAT in small areas. After correlation of the received data, five peaks are successfully identified with ray simulation. Layer-averaged water temperature of four layers in a vertical slice between two sound stations is calculated with inversion. Special attention is paid to correct the irregular station drifting. The inversion results showed great agreements with conductivity-temperature-depth (CTD) data, and its accuracy is further improved by the moving average.

The rest of the paper is structured as follows: In Section 2, the inversion method used to map the water temperature in the vertical slice is introduced. Experimental settings and ray simulation are also discussed. Section 3 shows the correlation of the received data, proposes the method to identify multi-arrival peaks, obtains the travel times of five ray paths with a focus on position correction and gives the sound speed and temperature profiling by inversion. The concluding remarks are given in Section 4.

## 2. Methods

In this section, the method of solving the inversion problem is introduced, and the experimental settings are described in detail. Finally, range independent ray simulation is conducted using temperature profiling and terrain data obtained by CTD casts. The results of the simulation will greatly help in the identification of the multi-path arrival peaks and vertical slice inversion.

### 2.1. Inversion Method

**Figure 1:**
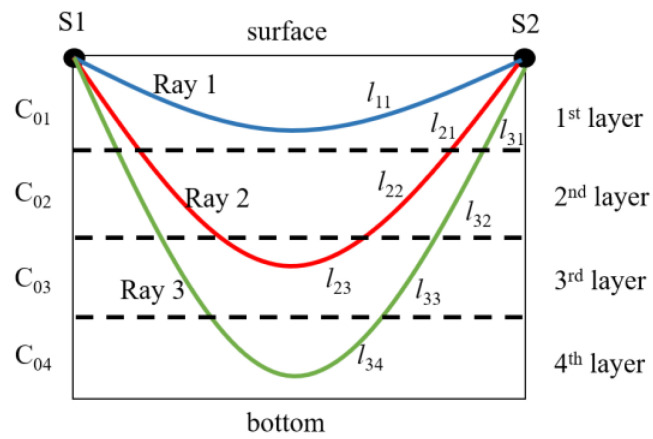
Schematic diagrams of ray paths across four layers between two stations; sound sources are deployed near the surface and negative sound velocity gradient is used here.

Prior to vertical slice inversion, two or more rays propagating across different layers should be identified. Supposing there are three rays across four layers between two stations, as shown in Figure 1, the travel times along each ray can be calculated as follows:

(1) $$\frac{l_{11}}{C_{01}+\delta C_{1}}=t_{01}+\delta t_{1}\;\frac{l_{21}}{C_{01}+\delta C_{1}}+\frac{l_{22}}{C_{02}+\delta C_{2}}+\frac{l_{23}}{C_{03}+\delta C_{3}}=t_{02}+\delta t_{2}\;\frac{l_{31}}{C_{01}+\delta C_{1}}+\frac{l_{32}}{C_{02}+\delta C_{2}}+\frac{l_{33}}{C_{03}+\delta C_{3}}+\frac{l_{34}}{C_{04}+\delta C_{4}}=t_{03}+\delta t_{3}$$

where *l_ij_* represents *i_th_* (*i* = 1, 2, 3) ray across *j_th_* (*j* = 1, 2, 3, 4) layer. *C_0j_* and *δC_j_* are the reference sound speed and its deviation of *j_th_* layer, respectively. *t_0i_* and *δt_i_* are the reference travel time and travel time deviation of *i_th_* ray, respectively.

The reference travel time of each ray can be formulated as follows:

(2) $$\frac{l_{11}}{C_{01}}=t_{01}\;\frac{l_{21}}{C_{01}}+\frac{l_{22}}{C_{02}}+\frac{l_{23}}{C_{03}}=t_{02}\;\frac{l_{31}}{C_{01}}+\frac{l_{32}}{C_{02}}+\frac{l_{33}}{C_{03}}+\frac{l_{34}}{C_{04}}=t_{03}$$

After taking the Taylor expansion under the assumption that *C_0_* ≫ *δC* of Equation (1), by neglecting the second and higher order terms of the Taylor expansion and combining Equation (2) [22], we obtain:

(3) $$-\frac{l_{11}}{C_{01}{}^{2}}\delta C_{1}=\delta t_{1}\;-\frac{l_{21}}{C_{01}{}^{2}}\delta C_{1}-\frac{l_{22}}{C_{02}{}^{2}}\delta C_{2}-\frac{l_{23}}{C_{03}{}^{2}}\delta C_{3}=\delta t_{2}\;-\frac{l_{31}}{C_{01}{}^{2}}\delta C_{1}-\frac{l_{32}}{C_{02}{}^{2}}\delta C_{2}-\frac{l_{33}}{C_{03}{}^{2}}\delta C_{3}-\frac{l_{34}}{C_{04}{}^{2}}\delta C_{4}=\delta t_{3}$$

The Equation (3) can be expressed as a matrix form:

(4) $$-\left[\begin{array}{cccc} \frac{l_{11}}{C_{01}{}^{2}} & 0 & 0 & 0\\ \frac{l_{21}}{C_{01}{}^{2}} & \frac{l_{22}}{C_{02}{}^{2}} & \frac{l_{23}}{C_{03}{}^{2}} & 0\\ \frac{l_{31}}{C_{01}{}^{2}} & \frac{l_{32}}{C_{02}{}^{2}} & \frac{l_{33}}{C_{03}{}^{2}} & \frac{l_{34}}{C_{04}{}^{2}} \end{array}\right]\left[\begin{array}{c} \delta C_{1}\\ \delta C_{2}\\ \delta C_{3}\\ \delta C_{4} \end{array}\right]=\left[\begin{array}{c} \delta t_{1}\\ \delta t_{2}\\ \delta t_{3} \end{array}\right]$$

Considering travel time errors **n**, the Equation (4) can be written as:

(5) y=Ex+n

where $y=\left\{\delta t_{i}\right\}$ is the travel time deviation vector, $x=\left\{\delta C_{j}\right\}$ is the sound speed deviation vector, and $E=\left\{-\frac{l_{ij}}{C_{0j}{}^{2}}\right\}$ is the coefficient matrix. Regularized inversion is used to solve the equation, where the optimal solution is:

(6) $$\hat{x}={\left(E^{T}E+\lambda H^{T}H\right)}^{-1}E^{T}y$$

*λ* is determined by making the square of errors ${\left\|\hat{n}\right\|}^{2}={\left\|y-E\hat{x}\right\|}^{2}$ smaller than a preset value (maximum acceptable inversion error). Moreover, *λ* is updated during the sound transmission process to make solutions much more flexible to trace the dynamic underwater environments. **H** is the regularization matrix constructed from the second-order derivative operator $\frac{\partial^{2}x}{\partial z^{2}}$, which is used to smooth the solution by the moving average of three consecutive layers [16]. Supposing there are four layers, **H** is expressed as:

(7) $$H=\left[\begin{array}{cccc} -2 & 1 & 0 & 0\\ 1 & -2 & 1 & 0\\ 0 & 1 & -2 & 1\\ 0 & 0 & 1 & -2 \end{array}\right]$$

Note that the dimensions of matrix **E** and **H** are determined by the number of identified rays and layers. To obtain a higher resolution of water temperature along the vertical slice, more rays are needed to decrease the linear dependence of each layer; the minimum number of identified rays is 2.

After obtaining the layer sound speeds, the corresponding temperatures can be calculated by Mackenzie’s formula for sound speed [23].

### 2.2. Experimental Settings

**Figure 2:**
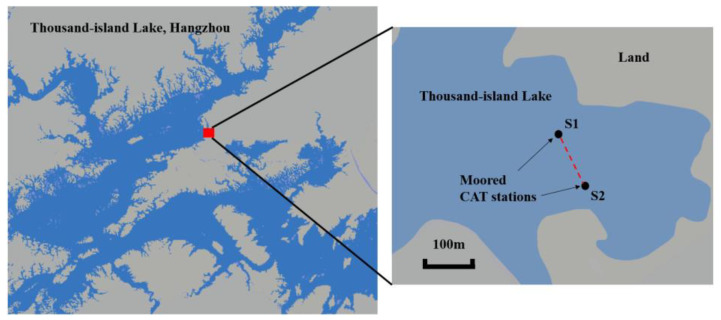
Experiment site in Thousand-Island Lake; two coastal acoustic tomography (CAT) systems are deployed from two fishing ships near the shore of Chun-an County, Hangzhou, China.

**Figure 3:**
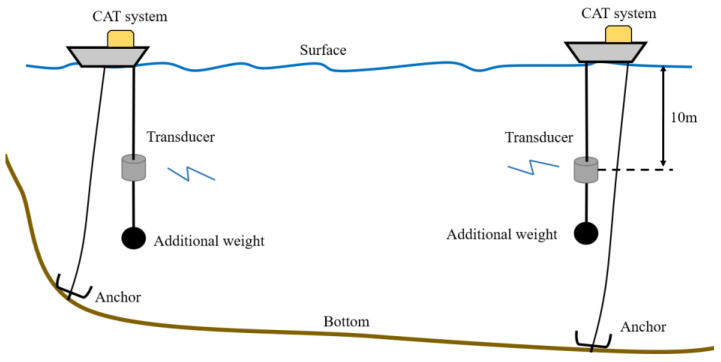
Experimental settings: two boat are fixed with an anchor, transducers are installed 10 m under the surface with additional weight, and CAT systems are synchronized with GPS.

The experiment is carried out in Thousand-Island Lake, Hangzhou, China on 12 November 2019, and experiment site is as shows in the Figure 2. Thousand-Island Lake has a distinct temperature stratification along the vertical slice with nearly no current, which is suitable for temperature measuring experiments. The experimental settings are displayed in Figure 3. Two stations with a distance of about 140 m are arranged in the lake to transmit and receive acoustic signals. The transducers are laid in the depth of 10 m from two fishing boats. The bottom of the experiment site has a thick sedimentary layer, which increases energy attenuation when sound waves interact with it. Furthermore, a 12 order M sequence with 8 repeats is used during the experiment to improve the signal-to-noise ratio (SNR) considering bottom reflection rays. Simultaneous transmission is not permitted as the distance between the two stations is too short. The round-robin transmission method, which means each station transmits signals in turns, is used in this experiment with a 90 s time delay. Additional weights are added in each transducer to assure the stability of the transducer position. CTD is used to measure the temperature profiling and depths of the experiment site. By sampling the depths along sound transmission line, rough terrain can be reconstructed using linear interpolation. According to pre-investigations, the terrain of Thousand-Island Lake does not have sheer variations; thus, the rough data can be used to simulate the ray transmission.

Other parameters used in this experiment are summarized in Table 1.

### 2.3. Ray Simulation

**Figure 4:**
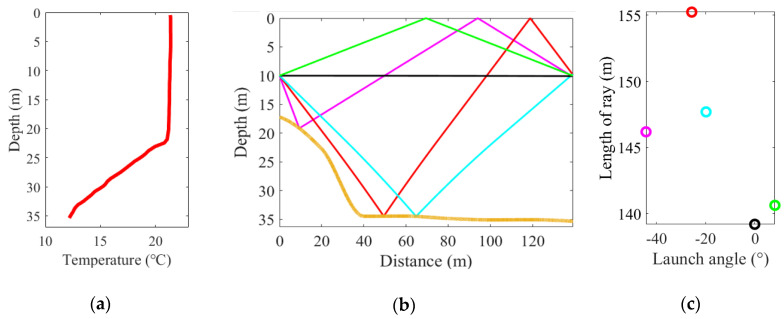
(**a**) Temperature profiling measured by CTD casts. (**b**) Ray simulation with five different ray paths. The yellow line represents the bottom terrain. (**c**) The launch angle of the rays and its corresponding lengths.

The temperature profiling is measured every 30 min by CTD casts and shows few variations, as shown in Figure 4a. As shown in the figure, the surface temperature is 21.3 °C and remains constant in the upper 20 m, which is mix layer. With depth increasing, there is a significant temperature decrease with linear change nearly being observed. At a depth of 35.17 m, the temperature reaches 12.17 °C.

With the help of temperature profiling and terrain data, ray paths can be simulated using Bellhop [24], as displayed in Figure 4b. Five rays with different paths are obtained with simulation, where the black ray is the direct path (D), the green ray is the surface reflect ray (S), the magenta ray is the first bottom-surface reflect ray (BS1), the cyan ray is the bottom reflect ray (B) and the red ray is the second bottom-surface reflect ray (BS2). These sound rays are numbered by length. Besides, the launch angle of rays and its corresponding length are diagramed in Figure 4c.

According to the temperature profiling, the vertical slice temperature field is divided into four layers: the first layer (0–20 m), the second layer (20–25 m), the third layer (25–30 m), and the fourth layer (below 30 m). By calculating the travel times of the rays across each layer, the reference travel time of each ray can be determined. The layer length of each ray and reference travel times are shown in Table 2. Note that this is not the only layer partition—other kinds of layer partitions are also performed during the inversion. The errors of the results are similar, which shows a low sensitivity of inversion to the layer partition. As a result, this paper only discusses the layer partition in Table 2.

## 3. Results

In this section, the correlation results are presented and the method to identify multi-arrival peaks is proposed. Besides, the significance of the station position drifting is discussed and the travel times along the five paths are corrected. After that, vertical slice inversion is performed using the travel times of the five rays. Abnormal data are eliminated by a predetermined temperature difference threshold, and the results are further improved by the moving average.

### 3.1. Correlation and Multi-Path Arrival Peak Identification

**Figure 5:**
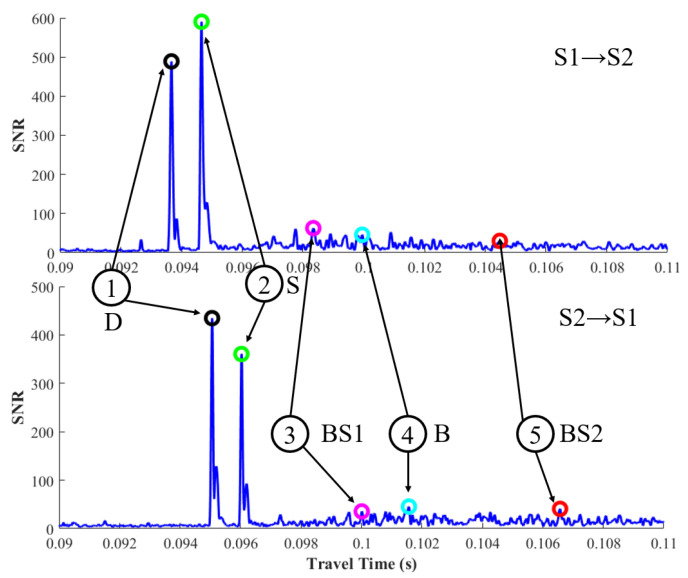
Correlation results for one transmission process. Upper and lower figures represent signals from station S1 to station S2 and station S2 to station S1, respectively.

**Figure 6:**
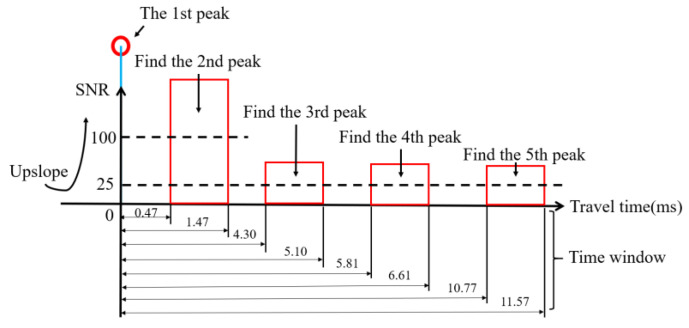
Multi-arrival peak identification method.

The correlation results of one sampled transmit–receive process are displayed in Figure 5. The five-colored circle marks the peaks of the corresponding arrival sound rays. As shown in Figure 5, the first and second peaks are significantly larger than the others, and it shows a great loss after bottom reflection. The first peak is not necessary the largest peak, thus, the upslope-point method, which uses a predetermined SNR threshold to find the first peak instead of the largest peak, is used during the arrival peak identification. The multi-arrival peaks are identified using time windows calculated by the reference travel time of each ray. The time windows are listed in Table 3. Note that every time window takes the first peak as the reference beginning instead of the prior peak. This is because the SNRs of the last three peaks are too small, which may be identified unsuccessfully during the identification process. The method to identify multi-arrival peaks is diagramed in Figure 6.

**Figure 7:**
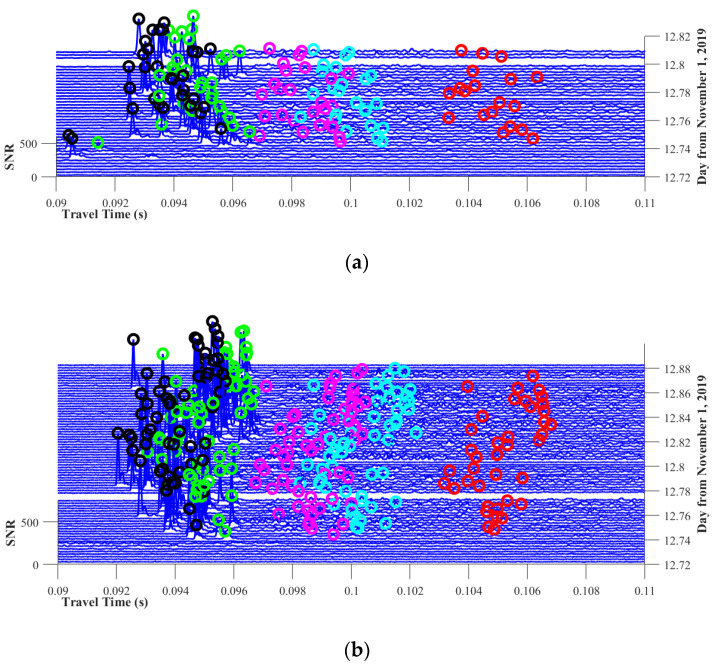
Stacked correlation data with five multi-arrival peaks being identified. (**a**) Correlation results from S1 to S2. (**b**) Correlation results from S2 to S1.

The multi-arrival peak identifications of all the data are stacked together and are shown in Figure 7. The figures are shown as three-dimension diagrams, which are displayed with an overlooking angle. The abscissa axis represents the travel times of each signal, the ordinate axis represents the time of signal sending and the height of the signal is the SNR value.

As shown in Figure 7, multi-arrival peaks are successfully identified using the method mentioned above. Unfortunately, station S1 missed some data after the experiment, which may be caused by incorrect operation of instruments. As a result, the number of signals in Figure 7a is smaller than in Figure 7b. In the inversion process, reciprocal transmission data are required to calculate δt, thus, some data from Figure 7b are not used. Moreover, it is clear that the travel time fluctuates significantly during the experiments.

### 3.2. Position Drifting and Travel Time Correction

**Figure 8:**
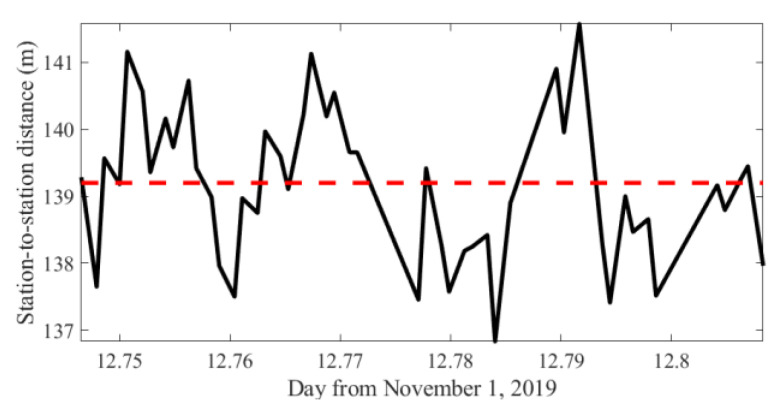
Station-to-station distance. Red dashed line represents the average distance.

According to the analysis in Section 3.1, irregular fluctuations of travel time can be observed in Figure 7. The fluctuations are a result from slight position drifting of the moored ships. Although the drifting may cause great errors, it can hardly be avoided, as transducers are hanging in water and are affected by turbulence and floating of the fishing boat. From Figure 4a,b, we can observe that the direct path can be regarded as a straight line which travels through the first layer that almost has no temperature variation, thus, the travel time of the direct path can accurately estimate the station-to-station distance. Using the first arrival time, the real-time station-to-station distance can be calculated, which is shown in Figure 8. It can be seen that the distances have intense fluctuations during the experiments. The root mean square error (RMSD) of the distance reaches 1.11 m.

The relative error of the range-average sound speed *C_m_* is expressed as [25]:

(8) $$\frac{\delta C_{m}}{C_{m}}=\frac{\delta L}{L}-\frac{\delta t_{m}}{t_{m}}$$

where *t_m_* is the mean travel time obtained from a pair of travel times at one time and *δt_m_* is its deviation. *L* and *δL* are the reference station-to-station distances and their deviations. The second term of the right side is eliminated by precise synchronization of GPS equipped in CAT systems. Thus, the deviation of distance become the main error of the sound speed calculation. In this experiment with a reference distance of 139.2 m, a reference sound speed of 1486.5 m/s and a distance deviation of 1.11 m, the expected sound speed deviation is 10.68 m/s. Under the conditions salinity *S* = 0.06 and temperature = 21.29 °C (data of the first layer), the relationship between the sound speed deviation *δC* and the temperature deviation *δT* is:

(9) δC=3.00δT

The 10.68 m/s sound speed deviations correspond to the 3.56 °C temperature deviations, which are unacceptable. Thus, correction of the position drifting must be performed before inversion. Under a reasonable assumption that the vertical current is negligible, the reciprocal travel times are calculated by the following equations [10]:

(10) $$t_{12}=\frac{L+\delta L}{C_{0}+\delta C_{m}+u_{m}-u_{2}}\approx \frac{\left(L+\delta L\right)}{C_{0}}\left\{1-\frac{\delta C_{m}+u_{m}-u_{2}}{C_{0}}\right\}\;t_{21}=\frac{L+\delta L}{C_{0}+\delta C_{m}+u_{m}-u_{1}}\approx \frac{\left(L+\delta L\right)}{C_{0}}\left\{1-\frac{\delta C_{m}+u_{m}-u_{1}}{C_{0}}\right\}$$

where *u_m_* is the range-average current, and *u_1_* and *u_2_* are the horizontal moving speed of S1 and S2, respectively. *T_12_* and *t_21_* are the travel times from S1 to S2 and S2 to S1, respectively. In this experiment, almost no current exists in the site, and the moving speed of the stations is also neglectable. As a result, Equations (10) are reduced to:

(11) $$t_{12}=\frac{\left(L+\delta L\right)}{C_{0}}\left\{1-\frac{\delta C_{m}}{C_{0}}\right\}\;t_{21}=\frac{\left(L+\delta L\right)}{C_{0}}\left\{1-\frac{\delta C_{m}}{C_{0}}\right\}$$

where the second item of the right side is caused by temperature deviations. Consequently, before the temperature inversion, travel time must be corrected as:

(12) $$t_{correct}=t\frac{\left(L+\delta L\right)}{L}$$

Equation (12) is used to correct all travel times under the assumption that all transmissions are affected by the same proportion.

**Figure 9:**
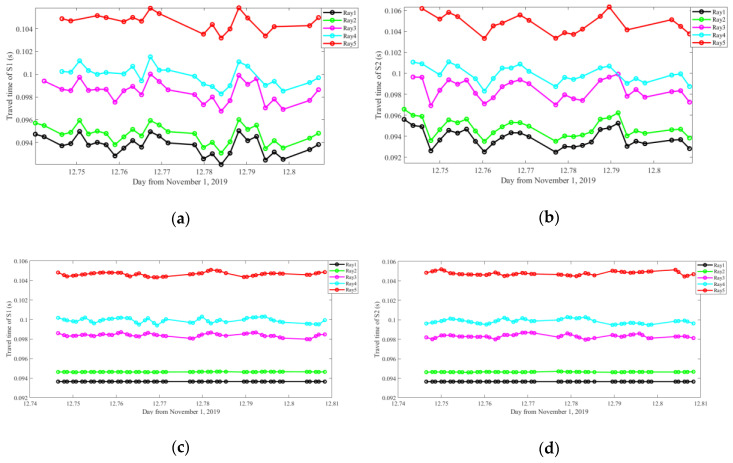
Travel times before and after the correction and interpolation. (**a**) and (**b**) are travel times obtained from S2 and S1 before the process. (**c**) and (**d**) are the corresponding travel times after the process.

Because the round-robin transmission is performed during the experiment, linear interpolation needs to be used to fill the missing data. Note that this process must be carried out after the travel time correction. Otherwise, the irregular drifting will bring big errors. The travel times of the sound waves between the two stations before and after the correction and interpolation are shown in Figure 9, which shows a smooth tendency as time goes by.

### 3.3. Inverted Temperature Profiling

According to Section 2.1, travel time deviations are required before the inversion, which can be calculated by:

(13) $$\delta t=\frac{\left(t_{12}+t_{21}-2t_{0}\right)}{2}$$

**Figure 10:**
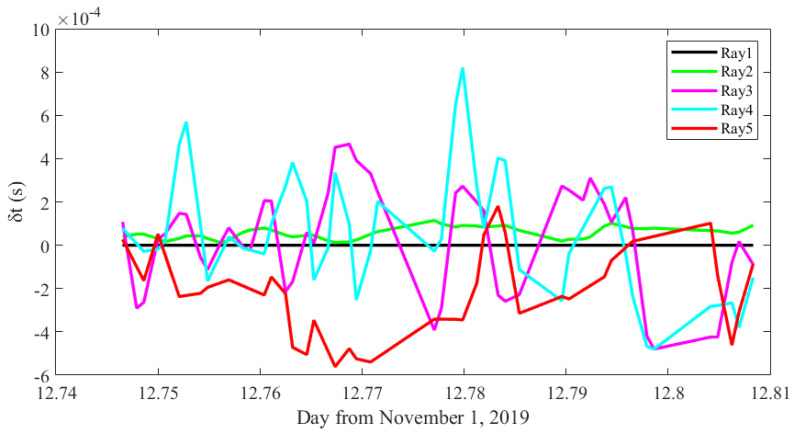
Travel time deviations.

The travel time deviations are diagramed in Figure 10. The travel time of ray 1 is used to correct other travel times, thus showing no deviation in the figure. The deviations of ray 2 show only slight variations, which corresponds to high SNRs of the second peaks. However, deviations of ray 3, ray 4, ray 5 have relative larger fluctuations, which also corresponds to the low SNRs of these peaks. The deviation fluctuations of the last three peaks are mainly caused by incorrect peak identifications. Different from the last three peaks, the SNR of the second peak is significantly larger, which is far less likely to be missed. As a result, slight variations in the second peak are assumed to result from the position drifting, which changes the ray path and is difficult to simulate.

**Figure 11:**
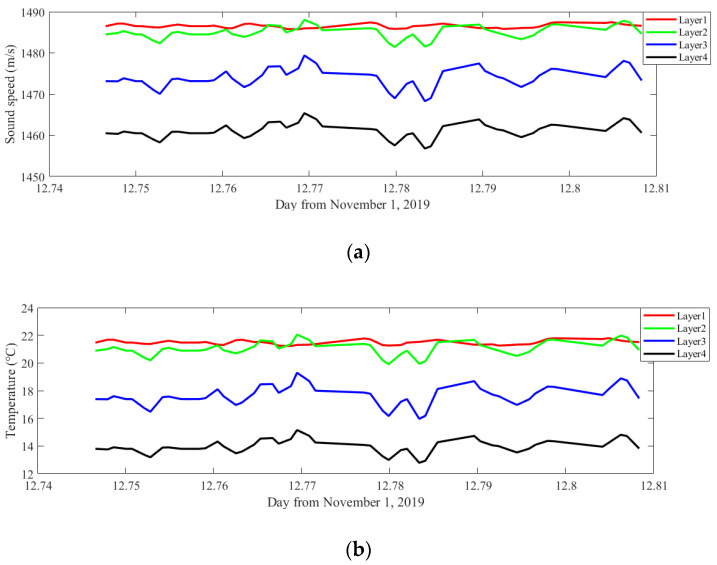
Inversion results. (**a**) Inverted sound speed profiling. (**b**) Inverted temperature profiling.

The inverted sound speed and temperature profiling are displayed in Figure 11. The sound speed and temperature have similar tendencies and both have a distinct stratification, which is consistent with CTD measurements. The temperature and sound speed of each layer remain stable although the layers 2, 3 and 4 show marked fluctuations.

**Figure 12:**
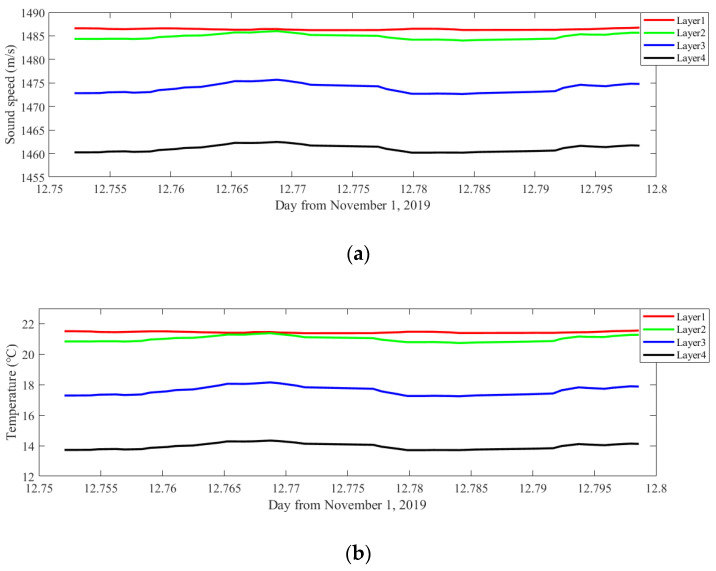
Inversion results after the moving average. (**a**) Sound speed profiling. (**b**) Temperature profiling.

Considering the short time intervals of the sound transmission used in the experiment, a moving average of 15 min data (10 ensembles) is performed to further increase the accuracy. The results after the moving average are diagramed in Figure 12.

After the moving average, the results are greatly smoothed and shows a constant stability. To better understand inverted results, their average sound speed and temperature and root mean square error (RMSE) during the experiment are listed in Table 4.

**Figure 13:**
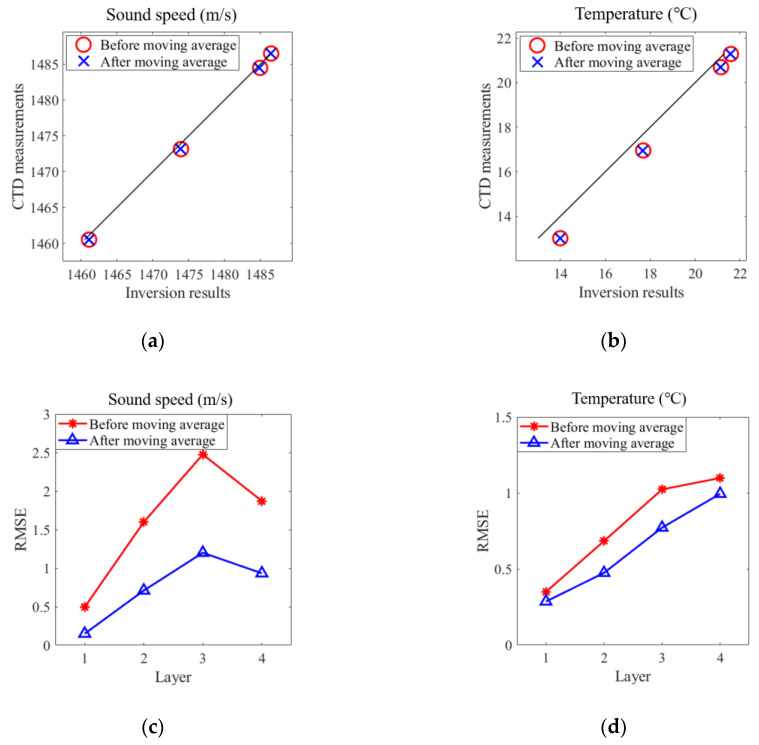
Regression plot and RMSE comparison. (**a**) Regression plot of the layer-average sound speed during the experiment. (**b**) Regression plot of the layer-average temperature during the experiment. (**c**) RMSE of the layer sound speed. (**d**) RMSE of the layer temperature.

It can be seen that the RMSEs of the sound speed and temperature are greatly improved after the moving average, while the average values change slightly. Consequently, the moving average is suitable to eliminate the noise and, at the same time, has a relatively small influence on results. More visible results are diagramed in Figure 13 using data from Table 4.

## 4. Discussion and Conclusions

In this study, two CAT systems were used to conduct a reciprocal sound transmission experiment in Thousand-Island Lake, China. CTD casts were performed during the experiment to obtain temperature profiling as well as terrain data. Range independent ray simulation was carried out to trace ray paths and calculate segment lengths when the sound rays propagate across each layer. Five rays across different layers and relevant travel times were obtained by sound simulation with Bellhop. Correlation of the received data with the transmitted signals was conducted for arrival peak identification. After that, the multi-path arrival peaks were distinguished. Five peaks were successfully identified, although the SNR of the first two peaks were significantly larger than the last three peaks. Time windows were used to determine the travel time for each ray path. The travel time of rays were extracted from the multi-arrival peaks and were preprocessed with a focus on position drifting correction. The layer sound speed and temperature were inverted by regularized inversion, showing a good consistency with the CTD measurements. RMSE of the temperature was 0.3494, 0.6838, 1.0236 and 1.0985 °C for layer 1, 2, 3 and 4, respectively, and it was further decreased to 0.2858, 0.4742, 0.7719 and 0.9945 °C, respectively, by a 15 min moving average.

This study shows a great feasibility and considerable accuracy of the high-frequency CAT system in short-range applications. Moreover, a relatively lager number of multi-path arrival peaks can be identified despite significant energy loss after bottom reflection, which increases the accuracy of vertical slice inversion. Additionally, 3D mapping of water temperature can be performed based on accurate vertical slice inversion along several transmission lines.

## Figures and Tables

**Table 1:** Parameters of experimental setting.

Item	Value
Central frequency	50 kHz
Transducer depth	10 m
Order of M sequence	12
Repeat number	8
Q ^1^	3
Station distance ^2^	140 m
Start and end time	17:30–21:00
round intervals ^3^	90 s

^1^ Q value is the number of cycles per digit in M sequence. ^2^ The distance is varied during the experiment. ^3^ Each station transmits signals every 3 min.

**Table 2:** Ray length and reference travel time.

Layer Length (m)	Ray 1	Ray 2	Ray 3	Ray 4	Ray 5	C_0_ (m/s)	T_0_ (°C)
Layer 1	139.20 ^1^	140.63	146.19	63.83	91.49	1486.5	21.29
Layer 2	0	0	0	30.64	22.73	1484.5	20.70
Layer 3	0	0	0	28.82	22.04	1473.2	16.95
Layer 4	0	0	0	24.41	18.98	1460.5	13.01
Total	139.20	140.63	146.19	147.69	155.23	\	\
Travel time ^2^(s)	0.093643	0.094606	0.098343	0.099852	0.104811	\	\

^1^ This distance is obtained by averaging the direct paths of all data. ^2^ The reference travel time of each ray, which may change as the station position changes.

**Table 3:** Time windows used in multi-arrival peak identification.

	Ray 1	Ray 2	Ray 3	Ray 4	Ray 5
Travel time(s)	0.093643	0.094606	0.098343	0.099852	0.104811
Time window ^1^(ms)	\	0.47–1.47	4.30–5.10	5.81–6.61	10.77–11.57
SNR ((Signal-to-noise ratio) threshold	100	100	25	25	25

^1^ The origin of the time windows is the position of the first peak.

**Table 4:** Average value and root mean square error (RMSE) of the results.

Item	Layer 1	Layer 2	Layer 3	Layer 4
**Average C ^1^ before MA ^2^**	1486.5	1485.0	1473.9	1461.1
**RMSE-C before MA**	0.4945	1.5984	2.4765	1.8721
**Average T ^3^ before MA**	21.60	21.16	17.69	13.99
**RMSE-T before MA**	0.3494	0.6838	1.0236	1.0985
**Average C after MA**	1486.4	1484.9	1473.9	1461.1
**RMSE-C after MA**	0.1502	0.7101	1.2004	0.9340
**Average T after MA**	21.57	21.13	17.67	13.98
**RMSE-T after MA**	0.2858	0.4742	0.7719	0.9945
**CTD-C ^4^**	1486.5	1484.5	1473.2	1460.5
**CTD-T ^5^**	21.29	20.69	16.95	13.01

^1^ C represents the sound speed (m/s); ^2^ MA represents the moving average; ^3^ T represents the temperature (°C); ^4^ The reference sound speed obtained from CTD; ^5^ The reference temperature obtained from CTD.

## References

[1] Wang L., Wang Y., Wang J., Li F. (2020). A High Spatial Resolution FBG Sensor Array for Measuring Ocean Temperature and Depth. Photonic Sens..

[2] Pivato M., Carniello L., Viero D.P., Soranzo C., Defina A., Silvestri S. (2020). Remote Sensing for Optimal Estimation of Water Temperature Dynamics in Shallow Tidal Environments. Remote Sens..

[3] Munk W., Wunsch C. (1979). Ocean acoustic tomography: A scheme for large scale monitoring. Deep Sea Res. Part A Oceanogr. Res. Pap..

[4] Munk W., Forbes A.M. (1989). Global Ocean Warming: An Acoustic Measure?. J. Phys. Oceanogr..

[5] Dushaw B.D., Worcester P.F., Munk W.H., Spindel R.C., Mercer J.A., Howe B.M., Metzger K., Birdsall T.G., Andrew R.K., Dzieciuch M.A. (2009). A decade of acoustic thermometry in the North Pacific Ocean. J. Geophys. Res..

[6] Zhao Z. (2016). Internal tide oceanic tomography. Geophys. Res. Lett..

[7] Worcester P.F., Lynch J.F., Morawitz W.M.L., Cornuelle B.D., Sutton P.J., Pawlowicz R. (1995). Evolution of the large-scale temperature field in the Greenland Sea during 1988–1989 from tomographic measurements. J. Acoust. Soc. Am..

[8] Skarsoulis E.K. (2001). Multi-section matched-peak tomographic inversion with a moving source. J. Acoust. Soc. Am..

[9] Yamoaka H., Kaneko A., Park J.H., Zheng H., Gohda N., Takano T., Zhu X.H., Takasugi Y. (2002). Coastal acoustic tomography system and its field application. IEEE J. Ocean. Eng..

[10] Kaneko A., Zhu X.H. (2020). Range-Average Measurement. Coastal Acoustic Tomography.

[11] Taniguchi N., Huang C.F., Arai M., Howe B.M. (2018). Variation of residual current in the Seto Inland Sea driven by sea level difference between the Bungo and Kii Channels. J. Geophys. Res. Ocean..

[12] Zhang K., Zhu X.H., Zhao R. (2018). Near 7-day response of ocean bottom pressure to atmospheric surface pressure and winds in the northern South China Sea. Deep Sea Res. Part I Oceanogr. Res. Pap..

[13] Zhu Z.N., Zhu X.H., Guo X., Fan X., Zhang C. (2017). Assimilation of coastal acoustic tomography data using an unstructured triangular grid ocean model for water with complex coastlines and islands. J. Geophys. Res. Ocean..

[14] Chen M., Kaneko A., Lin J., Zhang C. (2017). Mapping of a typhoon-driven coastal upwelling by assimilating coastal acoustic tomography data. J. Geophys. Res. Ocean..

[15] Huang H., Guo Y., Wang Z., Shen Y., Wei Y. (2019). Water Temperature Observation by Coastal Acoustic Tomography in Artificial Upwelling Area. Sensors.

[16] Zhang C., Kaneko A., Zhu X.H., Gohda N. (2015). Tomographic mapping of a coastal upwelling and the associated diurnal internal tides in Hiroshima Bay, Japan. J. Geophys. Res. Ocean..

[17] Syamsudin F., Chen M., Kaneko A., Adityawarman Y., Zheng H., Mutsuda H., Hanifa A.D., Zhang C., Auger G., Wells J.C. (2017). Profiling measurement of internal tides in Bali Strait by reciprocal sound transmission. Acoust. Sci. Technol..

[18] Zhang C., Kaneko A., Zhu X.H., Howe B.M., Gohda N. (2016). Acoustic measurement of the net transport through the Seto Inland Sea. Acoust. Sci. Technol..

[19] Fan W., Chenm Y., Pan H., Ye Y., Cai Y., Zhang Z. (2010). Experimental study on underwater acoustic imaging of 2-D temperature distribution around hot springs on floor of Lake Qiezishan, China. Exp. Therm. Fluid Sci..

[20] Li G., Ingram D., Kaneko A., Chen M., Gohda N., Polydorides N. (2017). Vertical underwater acoustic tomography in an experimental basin. J. Acoust. Soc. Am..

[21] Chen M., Syamsudin F., Kaneko A., Gohda N., Howe B.M., Mutsuda H., Dinan A.H., Zheng H., Huang C.F., Taniguchi N. (2018). Real-time offshore coastal acoustic tomography enabled with mirror-transpond functionality. IEEE J. Ocean. Eng..

[22] Kaneko A., Zhu X.H. (2020). Inversion on a vertical slice. Coastal Acoustic Tomography.

[23] Mackenzie K.V. (1981). Nine-term equation for sound speed in the oceans. J. Acoust. Soc. Am..

[24] Porter M.B. (2011). The Bellhop Manual and User’s Guide: Preliminary Draft.

[25] Liu W., Zhu X., Zhu Z., Fan X., Dong M., Zhang Z. A coastal acoustic tomography experiment in the Qiongzhou Strait. Proceedings of the 2016 IEEE/OES China Ocean Acoustics (COA).

